# Alternariol Detoxification by *Rhodotorula taiwanensis* P1 Isolated from a Pear and Its Mechanism

**DOI:** 10.3390/toxins18090367

**Published:** 2026-08-26

**Authors:** Gyu-Mi Jung, Sung-Yong Hong, Ae-Son Om

**Affiliations:** Department of Food and Nutrition, College of Human Ecology, Hanyang University, Seoul 04763, Republic of Korea; mb3159@hanyang.ac.kr (G.-M.J.); lunohong@hanyang.ac.kr (S.-Y.H.)

**Keywords:** *Alternaria*, alternariol, detoxification, yeast, *Rhodotorula taiwanensis*

## Abstract

Alternariol (AOH) is a mycotoxin produced mainly by *Alternaria alternata* on fruits including tomatoes and strawberries as well as cereal grains including wheat and soybeans. In this study, we isolated *Rhodotorula taiwanensis* (*R. taiwanensis*) P1 from a pear and investigated the effects of incubation time and temperature on AOH reduction rates and the mechanism involved in AOH detoxification by the yeast strain. The yeast strain showed a 76.10% AOH reduction rate from the initial level (1 μg/mL) at 30 °C after 48 h of incubation. The yeast cell-free filtrate from the yeast culture did not increase the AOH reduction rate relative to the control without cell-free filtrate after 48 h. In addition, the AOH reduction rate by heat-inactivated yeast cells (74.57%) was similar to that by viable yeast cells (76.33%). The AOH detoxification test using the yeast cell wall fraction showed that AOH binds to its cell walls and that approximately 1/3 of the AOH bound onto the cell walls was extracted from them. These data strongly suggest that the AOH detoxification by the yeast strain was not due to degradation by either intracellular enzymes or extracellular enzymes of the yeast culture but was due to binding to yeast cell walls. The use of spheroplasts, in which cell walls are deficient, confirmed that AOH detoxification occurred by binding onto the cell walls of *R. taiwanensis*. Our data demonstrated that *R. taiwanensis* P1 was able to remove AOH by adsorption onto its cell walls. These results could help in the development of potential strategies to effectively mitigate AOH contamination of food.

## 1. Introduction

Alternariol (AOH) is a toxic secondary metabolite produced mainly by *Alternaria alternata* on fruits including tomatoes, apples, and strawberries as well as cereal grains including wheat, sorghum, and soybeans [1,2,3]. Although most *Alternaria* toxins exhibit low levels of acute toxicity, AOH has been reported to exhibit mutagenic, cytotoxic, carcinogenic, and genotoxic properties on human and animal cells [2,3,4,5,6]. It is also known that AOH is linked to squamous cell cancer in human esophageal tissues during early development [7]. Since there is a scarcity of comprehensive data on AOH toxicity and its human exposure, the legal limits for food were not set [8]. However, a toxicological assessment conducted by the European Food Safety Authority (EFSA) identified *Alternaria* toxins including AOH as significant public health threats [9]. Recently, the European Commission (EC) has recommended specific guidance levels for three main *Alternaria* toxins (AOH, alternariol monomethyl ether [AME], and tenuazonic acid [TeA]) in certain foods, including 10 μg/kg of AOH for processed tomato products [10].

Similar to other mycotoxins, AOH is relatively thermal stable during food processing, leading to difficult elimination of AOH in food products [2]. Multiple approaches have been proposed to detoxify AOH, including physical decontamination such as binding to cyclodextrin polymers, chemical methods such as breakdown by ozone treatments, and biological decontamination methods such as inhibition of fungal growth or toxin production by microbial antagonists and biodegradation by microorganisms or enzymes [2,11,12,13,14]. However, physical and chemical decontamination strategies for AOH detoxification have several practical limitations. Both methods can have detrimental impacts on food quality and nutritional value [2]. Thus, biological control methods provide a highly promising alternative approach.

To date, few studies on AOH-detoxifying microorganisms have been documented [12,13]. The cotA laccase from a *Bacillus licheniformis* strain was able to degrade 96.56% of AOH at 37 °C after 24 h [13]. The optimal temperature and pH for AOH degradation by the enzyme were 80 °C and pH 9. In another study, heat-inactivated dried powder of a *Saccharomyces cerevisiae* strain was capable of removing 56.3% of the initial AOH level (1 μg/mL) after 24 h [12]. The optimal pH for AOH removal by the inactivated powder was pH 9. Hence, in this study we isolated an AOH-detoxifying yeast, *Rhodotorula taiwanensis* (*R. taiwanensis*) P1, from a pear, found the optimal temperature and pH for AOH detoxification by the yeast strain, and investigated the mechanism involved in AOH detoxification.

## 2. Results and Discussion

### 2.1. Validation of HPLC-UVD Method for Quantification of AOH

**Figure 1:**
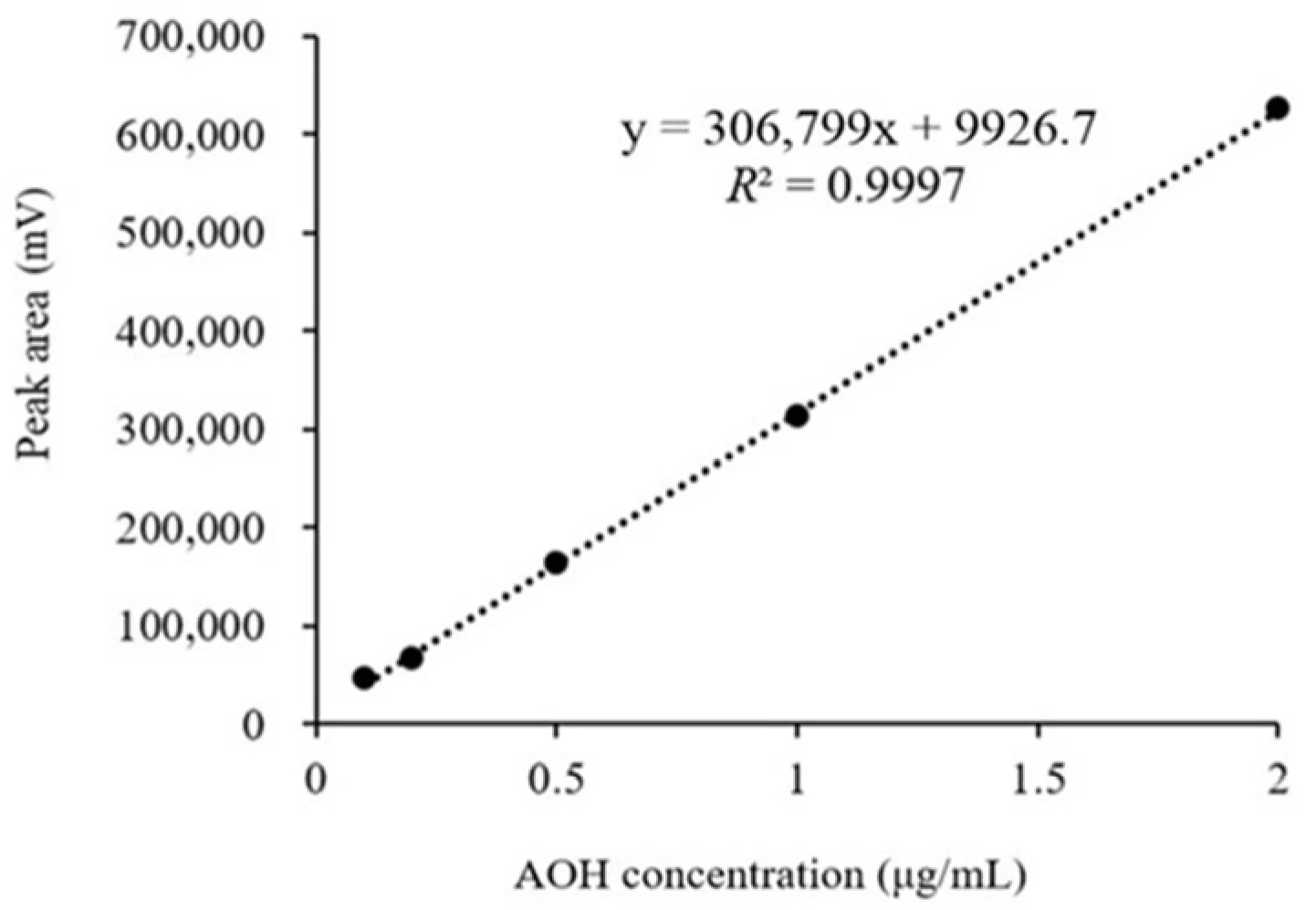
Calibration curve of AOH standards for AOH quantification. After preparation of a series of AOH standards (0.1, 0.2, 0.5, 1.0, and 2.0 μg/mL), each standard was injected into HPLC-UVD in triplicate.

The high-performance liquid chromatography-UV detector (HPLC-UVD) method for AOH quantification was validated for three evaluation metrics, namely linearity, precision, accuracy, and sensitivity. To assess the linearity of the standard curve, linear regression analysis was used with five levels of AOH standard solutions. The r^2^ value of the curve was 0.999 (Figure 1). Thus, we concluded that the standard curve was linear over the range of 0–2 μg/mL AOH.

To evaluate the accuracy of the analytical method, we assessed the recovery of AOH extracted from 0.1 M KH_2_PO_4_ buffer solution (pH 8) spiked with three concentrations of AOH standard solutions (0.2, 1, and 2 μg/mL). The recovery rates for AOH in phosphate buffer solution (pH 8) were in the range of 92.01–100.33% (Table 1). The recoveries were above 70% in all of the samples, which is consistent with the recovery rates (70–125%) recommended by the Association of Official Analytical Chemists (AOAC) for food samples with a mycotoxin contamination level of 10 μg/kg, although the recovery range for AOH was not established [15]. Hence, it was concluded that the analytical method yielded excellent recoveries of AOH in phosphate buffer solution (pH 8).

To determine the precision of the analytical method, we analyzed within-day precision (repeatability). The relative standard deviation (*RSDr*), which was derived from the extraction of AOH in phosphate buffer solution (pH 8), ranged from 1.99 to 8.04% (Table 1). Those values were in agreement with the reference level (below 15%) recommended by AOAC for food samples with a mycotoxin contamination level of 10 μg/kg [15]. Thus, we concluded that the HPLC-UVD method showed excellent precision in quantification of AOH in phosphate buffer solution (pH 8).

**Figure 2:**
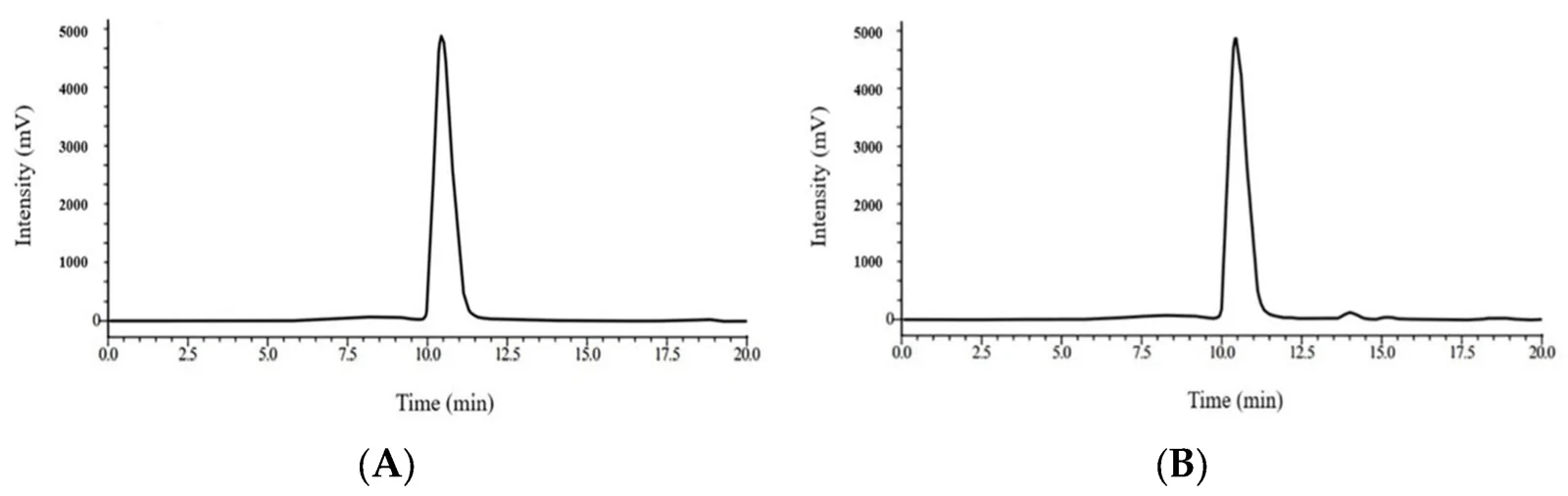
HPLC-UVD chromatograms of AOH. Chromatograms of (**A**) AOH standard solution (0.5 μg/mL) and of (**B**) AOH extracted from 0.1 M KH_2_PO_4_ buffer solution (pH 8). The AOH peak exhibited a retention time (RT) of 10.6 min.

To evaluate the sensitivity of the HPLC-UVD method, we determined a limit of quantification (LOQ) and limit of detection (LOD). The LOQ and LOD for AOH were 0.068 and 0.023 μg/mL, respectively. These values were sufficiently low to detect trace amounts of AOH. This indicates that the method has high sensitivity for quantification of AOH in phosphate buffer solution (pH 8). Typical chromatograms of AOH in a standard and AOH extracted from phosphate buffer solution (pH 8) are shown in Figure 2.

### 2.2. Isolation of Yeasts from Fruits and Identification of Isolated Yeast Strains

**Figure 3:**
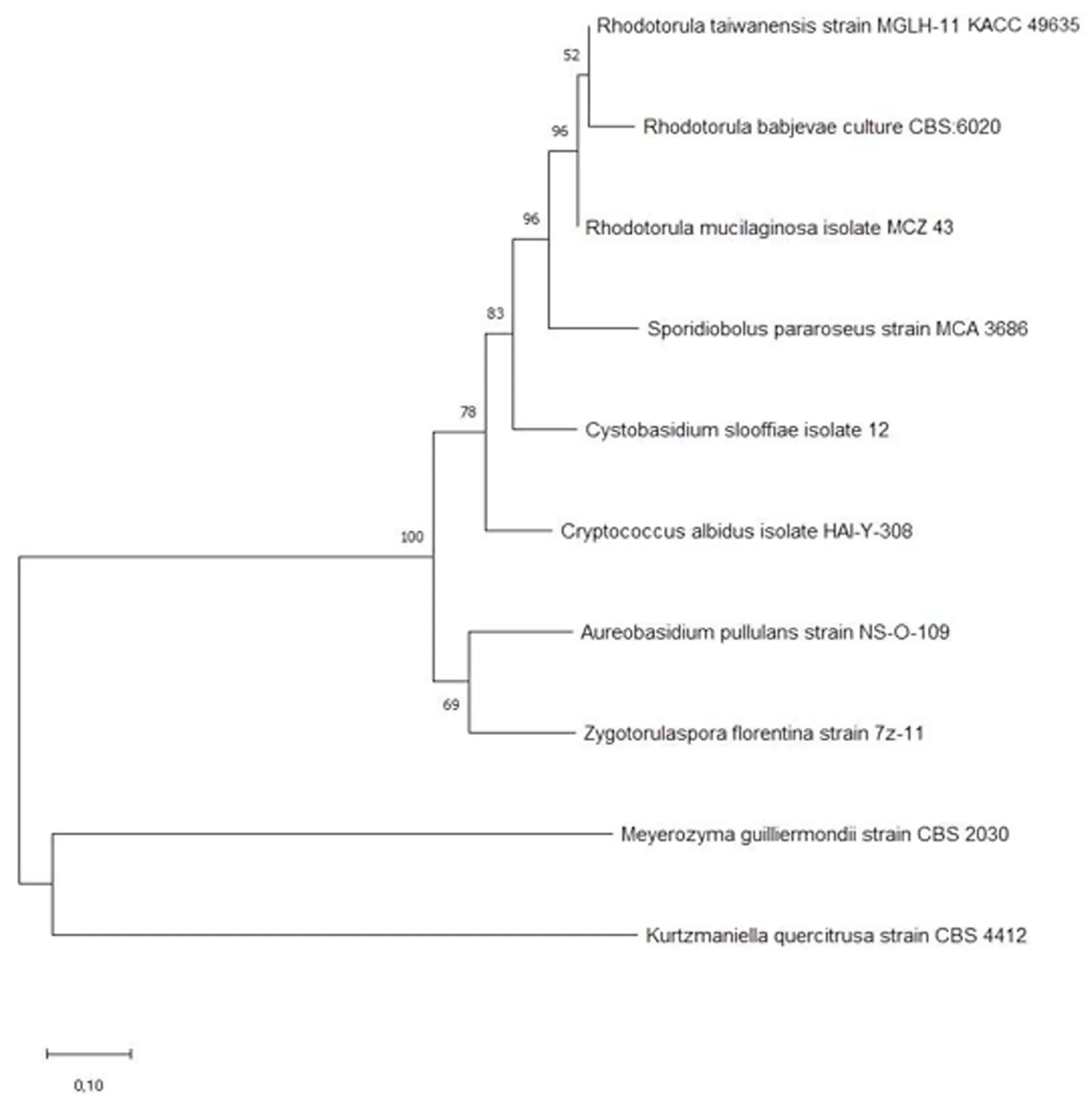
Phylogenetic tree based on ITS1-5.8S rDNA-ITS2 sequence in genomic DNA of 10 yeast strains isolated from pears, grapes, and strawberries. The neighbor-joining (NJ) method was employed to construct the phylogenetic tree.

In total, 10 yeasts were isolated from three types of fruits (pears, grapes, and strawberries). Genetic identification was carried out on all 10 isolated yeasts, based on the internal transcribed spacer 1 (ITS1)-5.8S rDNA-ITS2 region on yeast rDNA. A polymerase chain reaction (PCR) successfully amplified the ITS-5.8S rDNA region on yeast genomic DNA from all 10 yeast isolates. Analyses using the nucleotide Basic Local Alignment Search Tool (BLASTn, https://blast.ncbi.nlm.nih.gov/, accessed on 5 April 2024) showed high sequence similarity, ranging from 99.20% to 99.98%, between the yeast isolates and the database in the National Center for Biotechnology Information (NCBI). (Table 2). The data showed that five of the 10 yeast strains were isolated from pears, whereas three and two yeast strains were from grapes and strawberries, respectively. They also revealed that different yeast strains were isolated from different types of fruits, although one *Rhodotorula* strain (*R. mucilaginosa*) was isolated from a grape instead of a pear (Table 2). When we grouped the yeast isolates, those from pears (cultivar Soojeong) belonged to four orders (Sporidiobolales, Serinales, Cystobasidiales, and Dothideales), while those from grapes (cultivar Campbell Early) and strawberries (cultivar Seolhyang) belonged to two orders for each type of fruit (Saccharomycetales and Sporidiobolales for grapes, Sporidiobolales and Tremellales for strawberries). This indicates that yeast strains from pears further belong to the phyla Basidiomycota, Saccharomycota, and Ascomycota, whereas those from grapes belong to the phyla Ascomycota and Basidiomycota, and those from strawberries are members of Basidiomycota. These results are slightly different from those from other earlier studies [16,17,18]. One previous study from Lithuania reported that *Hanseniaspora* sp., *Metschnikowia* sp., *Aureobasidium* sp., and *Pichia* sp. were prevalent species isolated from pears (*Pyrus communis*) [16]. Also, one study from Germany documented that 23 yeast species were isolated from 10 different grape varieties in Germany, such as *Rhodosporidium* sp., *Hanseniaspora* sp., *Metschnikowia* sp., *Filobasidium* sp., *Sporidiobolus* sp., *Cryptococcus* sp., *Rhodotorula* sp., *Zygotorulapspora* sp., and *Candida* sp [17]. Another previous study described that *Rhodotorula* sp., *Pichia* sp., and *Metschnikowia* sp. were commonly found in strawberries (cultivar Elsanta) [18]. This discrepancy could have been due to differences in regional climate or cultivars of pears, grapes, and strawberries. Figure 3 displays the phylogenetic tree using 10 yeast isolates.

### 2.3. Selection of AOH-Detoxifying Yeasts

In order to evaluate AOH reduction rates by the yeast isolates, we cultured the 10 isolates in 0.1 M KH_2_PO_4_ buffer solution (pH 8) spiked with 1 μg/mL of AOH at 30 °C for 48 h. The AOH reduction rates from four out of the 10 yeast strains were in the range from 21.73% to 76.87% (Table 3). *R. taiwanensis* P1, isolated from a pear, showed the highest AOH reduction rate (76.87%; 53.50% yeast-specific reduction rate after subtraction of the background loss), followed by *R. babjevae* P2 (53.40%; 30.03% yeast-specific reduction rate after subtraction of the background loss), *Kurtzmaniellla quercitrusa* P3 (37.77%; 14.40% yeast-specific reduction rate after subtraction of the background loss), and *M. guilliermondii* G1 (31.83%; 8.46% yeast-specific reduction rate after subtraction of the background loss) isolated from a grape. The other strains produced similar AOH reduction rates to the control without yeasts (23.37%), indicating that they were not able to detoxify AOH. Thus, *R. taiwanensis* P1 (GenBank accession number PZ794421), which belongs to Sporidiobolales (Basidiomycota), was selected for this study.

### 2.4. Impacts of Different Culture Conditions on AOH Reduction Rates by R. taiwanensis P1

**Figure 4:**
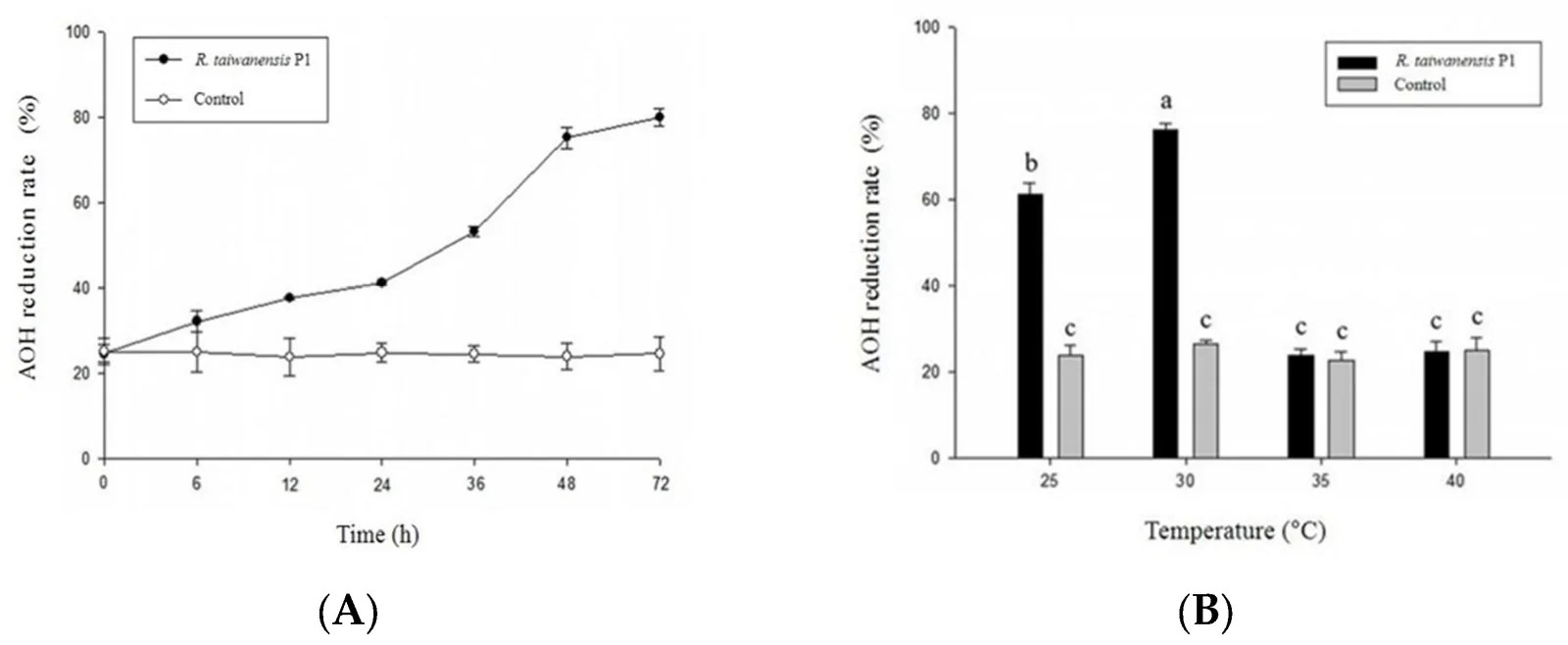
Impact of different environmental parameters on AOH reduction rates by *R. taiwanensis* P1 in 0.1 M KH_2_PO_4_ buffer solution (pH 8) spiked with 1 μg/mL of AOH under agitation at 150 rpm. (**A**) Impact of incubation time on AOH reduction rates at 30 °C for 72 h and (**B**) impact of incubation temperature on AOH reduction rates after 48 h. The AOH reduction rates were obtained in triplicate and are represented as the mean ± standard deviation. Different letters indicate significant differences between data (*p* < 0.05).

Since some key environmental factors such as incubation temperature and time influence AOH reduction rates, we evaluated the impact of different abiotic parameters on AOH reduction rates by *R. taiwanensis* P1. When *R. taiwanensis* P1 was incubated in 5 mL of 0.1 M KH_2_PO_4_ buffer solution (pH 8) spiked with 1 μg/mL of AOH during 72 h, AOH reduction rates increased with increasing incubation time (Figure 4A). The yeast strain produced the highest AOH reduction rate at 72 h (79.87%; 55.24% yeast-specific reduction rate after subtraction of the background loss), and the second highest AOH reduction rate was at 48 h (75.23%; 51.23% yeast-specific reduction rate after subtraction of the background loss), although there was no statistically significant difference between them. Also, our data showed a rapid increase in the AOH reduction rate between 24 and 48 h, which is slightly different from one previous study [12]. Wang and co-workers described a sharp increase in the AOH reduction rate during early-stage incubation (between 0 and 15 h), with the maximum at 50 h (approximately 100%) when they treated 1 μg/mL of AOH solution with a heat-inactivated *Saccharomyces cerevisiae* strain [12]. This discrepancy could have resulted from the use of a different yeast strain or dead yeast cells. Next, to assess the impact of incubation temperature on AOH reduction rates by the yeast strain, the strain was incubated in 5 mL of 0.1 M KH_2_PO_4_ buffer solution (pH 8) spiked with 1 μg/mL of AOH at four different temperatures (25, 30, 35, and 40 °C) for 48 h. The AOH reduction rates were highest (76.10%; 49.47% yeast-specific reduction rate after subtraction of the background loss) at 30 °C, which was followed by 25 and 35 °C (or 40 °C) (Figure 4B). These results may have been due to changes in the cell walls of *R. taiwanensis* P1 and its growth decrease above 30 °C. One previous study reported that when *S. cerevisiae*, which was cultured at 30 °C, was shifted to 42 °C incubation for 1 h, the heat shock increased the stiffness of the yeast cell wall and resulted in cell cycle arrest [19]. Since our data showed that *R. taiwanensis* P1 reduced the high level of AOH significantly after 48 h at 30 °C, we chose this incubation condition for further experiments.

### 2.5. Mechanism of AOH Detoxification by R. taiwanensis P1

**Figure 5:**
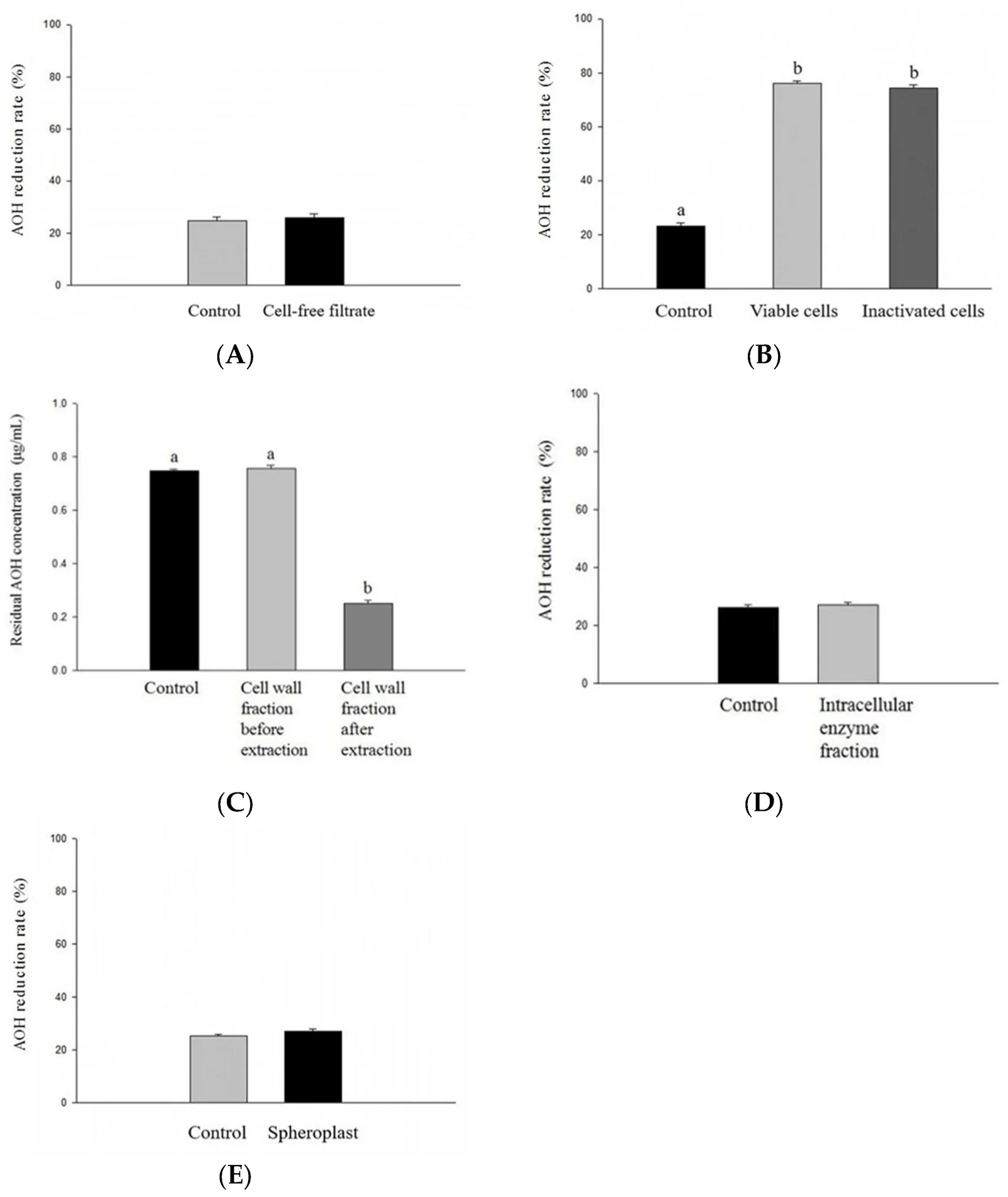
AOH reduction rates in 1 μg/mL of AOH solution after treatments with different cell fractions of *R. taiwanensis* P1 under agitation at 150 rpm after 48 h at 30 °C. (**A**) Yeast cell-free filtrate, (**B**) viable or heat-inactivated yeast cells, (**C**) cell wall fraction, (**D**) intracellular enzyme fraction, and (**E**) yeast spheroplasts. The AOH removal rates were obtained in triplicate and are represented as mean ± standard deviation. Different letters indicate significant differences between data (*p* < 0.05).

The AOH detoxification by *R. taiwanensis* P1 could occur by one of three mechanisms: (1) AOH degradation by extracellular enzymes from the yeast strain, (2) adsorption onto the yeast cell walls, and (3) AOH biodegradation by intracellular enzymes inside the yeast cells. Thus, we first analyzed AOH reduction rates using yeast cell-free filtrates. The AOH reduction rate by yeast cell-free filtrate from *R. taiwanensis* P1 culture in phosphate buffer solution (pH 8) was similar to that by the control without cell-free filtrate after 48 h incubation (25.93% for the cell-free filtrate, 24.60% for the control), indicating that the yeast cell-free filtrate did not play any role in AOH degradation (Figure 5A). Zhu and collaborators reported that the patulin reduction rate by extracellular culture filtrate of *Rhodosporidium paludigenum*, which belongs to the same phylum Basidiomycota as *Rhodotorula* sp., did not show any difference from that of control lacking the yeast strain (approximately 10%) after 48 h incubation. This result is in line with our results, although their study used a different mycotoxin and yeast strain [20].

Also, one earlier study described that heat-inactivated *S. cerevisiae* had significant AOH reduction rates [12]. Thus, we compared the AOH reduction rate by viable *R. taiwanensis* P1 with that by heat-treated yeast cells (dead cells) after autoclaving the viable yeast cells at 121 °C for 15 min. The autoclaved yeast cells showed a 74.57% AOH reduction rate after 48 h incubation, which was similar to the AOH reduction rate by the viable yeast cells (76.33%) (Figure 5B). These results are in accordance with those from the earlier study, although it used a different yeast strain [12]. Wang and collaborators documented that a heat-inactivated *S. cerevisiae* strain produced a 56.3% AOH reduction rate after 24 h incubation when they used 1 μg/mL of AOH solution [12]. In addition, some earlier studies reported that patulin reduction by activated or inactivated *S. cerevisiae* was attributed to adsorption onto its cell walls [21,22,23]. In our study, the similar AOH reduction rates between viable cells and heat-inactivated cells of *R. taiwanensis* P1 indicate that the AOH reduction could have been attributed to physical binding of AOH onto its cell walls. Thus, after disrupting *R. taiwanensis* P1 cells by grinding under liquid N_2_ and isolating the yeast cell walls, we treated the cell walls with 1 μg/mL of AOH solution for 48 h and extracted the residual AOH from them. HPLC-UVD analysis showed that of the 0.78 μg/mL of AOH, which was bound onto the cell walls, 0.24 μg/mL of residual AOH (approximately 1/3 of AOH on the cell walls, extraction efficiency = 31%) was extracted from the yeast cell walls (Figure 5C). In addition, when we incubated 1 μg/mL of AOH solution for 48 h after addition of intracellular enzymes isolated from *R. taiwanensis* P1, the AOH reduction rate was similar to the control without the intracellular enzyme fraction (27.19% for the intracellular enzyme fraction, 26.28% for the control) (Figure 5D). These data suggest that AOH was removed by adsorption onto the cell walls of *R. taiwanensis* P1 and that the AOH binding onto the cell walls is partly reversible. Guo and co-workers reported that there was no statistically significant difference between viable and heat-treated *S. cerevisiae* in patulin removal and that 35.05% of patulin was removed by its cell wall fraction [21], which is consistent with our results although we used a different mycotoxin and yeast strain. In order to confirm that intracellular enzymes play no role in AOH reduction, we analyzed AOH reduction rates using yeast spheroplasts, which lack cell walls. The AOH reduction rate by spheroplasts of *R. taiwanensis* P1 was 26.37%, which was similar to that from the control without spheroplasts (25.43%) (Figure 5E). When taken together, our data strongly suggest that AOH reduction by *R. taiwanensis* P1 was due to binding onto the yeast cell walls.

AOH contamination in fruits such as tomatoes and strawberries as well as cereal grains poses a health risk to the public. Development of AOH decontamination strategies using yeast strains has gained significant interest. In the current study, we isolated *R. taiwanensis* P1 from a pear as an AOH-detoxifying yeast strain and investigated its AOH detoxification mechanism. Our results showed that some environmental factors such as incubation temperature and time influenced the AOH reduction rate by *R. taiwanensis* P1. The yeast strain was able to detoxify approximately 80% of AOH after 72 h at 30 °C. In addition, AOH was not reduced by intracellular enzyme fractions or spheroplasts of the yeast strain, whereas it was reduced by yeast cell wall fractions. This suggests that AOH was bound onto the cell walls of *R. taiwanensis* P1. Guo and colleagues described that in patulin binding to heat-treated *S. cerevisiae*, hydrophobic interactions between patulin and cell wall components play a major role [21]. In our study, it is likely that weak non-covalent hydrophobic interactions were involved in the AOH binding processes onto the cell walls of *R. taiwanensis* P1, considering the partial release of AOH from its cell walls. Future studies will reveal the detailed mechanisms of AOH binding onto cell wall components of *R. taiwanensis* P1.

## 3. Conclusions

The current study aimed to investigate the effects of incubation time and temperature on AOH reduction rates and the mechanism involved in AOH detoxification by *R. taiwanensis* P1 after its isolation from a pear. Our data showed that the yeast strain was able to detoxify 76.10% of the initial AOH level (1 μg/mL) at 30 °C after 48 h incubation. In particular, our data demonstrated that AOH reduction rates were not increased by yeast cell-free filtrate, intracellular enzymes, or spheroplasts compared to control groups. This suggests that the AOH detoxification by the yeast strain was not due to degradation by either intracellular enzymes or extracellular enzymes of the yeast culture, but rather was due to adsorption onto yeast cell walls. Moreover, approximately 1/3 of AOH bound to the cell walls was extracted, which supports that AOH was adsorbed onto yeast cell walls. The present study could help remove AOH produced by *Alternaria* spp. on food such as fruits.

## 4. Materials and Methods

### 4.1. Chemicals and Reagents

AOH standard (≥96.0% purity), zinc sulfate monohydrate (ZnSO_4_·H_2_O, ≥99.0% purity), lyticase, disodium ethylenediaminetetraacetic acid (Na_2_EDTA), tetracycline, and chloramphenicol were purchased from Sigma-Aldrich Co., Ltd. (St. Louis, MO, USA), while isopropanol and anhydrous sodium sulfate (Na_2_SO_4_) were from Junsei Chemical Co., Ltd. (Tokyo, Japan). HPLC-grade methanol (MeOH, ≥99.99% purity), potassium acetate, and sodium bicarbonate (NaHCO_3_) were obtained from Samchun Chemical Co., Ltd. (Seoul, Republic of Korea), while D-sorbitol and ethyl acetate were from Daejung Chemical Co., Ltd. (Seoul, Republic of Korea). Sodium dodecyl sulfate (SDS) and Tris base were purchased from Bio-Rad (Hercules, CA, USA).

### 4.2. Isolation of Yeasts from Fruits

Three types of fresh fruits (pears, grapes, and strawberries) were collected from different orchards (Yangpyeong and Gapyeong in Gyeonggi-do, Jincheon in Chungcheongbuk-do) in South Korea and used to search for AOH-detoxifying yeasts. To collect the surface microorganisms, the fruits were swabbed with sterile cotton swabs and the microorganisms on the swabs were suspended in sterile saline. Subsequently, they were inoculated onto potato dextrose agar (PDA; MB Cell, Seoul, Republic of Korea) plates containing both tetracycline and chloramphenicol (0.5 μg/mL for each). After the PDA plates were incubated at 30 °C for 3 days, yeast colonies were transferred onto yeast extract peptone dextrose (YPD; MB Cell, Seoul, Republic of Korea) agar plates. Then, they were further incubated at 30 °C for 3 days and were pure-isolated by a streak plate method.

### 4.3. Identification of Yeast Isolates

Yeast genomic DNA was isolated according to the small-scale miniprep protocol described in a Cold Spring Harbor Laboratory manual with slight modifications [24]. Briefly, each yeast isolate was cultured in 5 mL of YPD at 30 °C under agitation at 150 rpm for 2 days. After the cells were centrifuged at 2000 rpm for 5 min and the supernatant was discarded, the yeast cell pellet was resuspended in 0.5 mL sorbitol solution containing 0.1 M Na_2_EDTA (pH 7.5) and 1 M sorbitol. Subsequently, 0.02 mL of lyticase (0.5 mg/mL) was added to the yeast cell suspension, and it was incubated at 37 °C for 1 h. After the cells were centrifuged at 2000 rpm for 1 min and the supernatant was discarded, the yeast cell pellet was resuspended in 0.05 mL of 10% SDS. Then, 0.5 mL of 50 mM Tris·Cl (pH 7.4) buffer solution containing 20 mM Na_2_EDTA (pH 7.5) was added to the yeast cell suspension, and it was incubated at 65 °C for 30 min. After 0.2 mL of 5 M potassium acetate was added to the yeast cell suspension, it was further incubated on ice for 1 h. Finally, after centrifugation at 2000 rpm for 5 min, an equal volume of 100% isopropanol was added to the supernatant, and it was incubated at room temperature for 10 min. The air-dried DNA pellet was resuspended in 0.3 mL of TE (pH 7.4) after centrifugation at 2000 rpm for 5 min.

Yeast isolates were identified by DNA sequencing of the ITS1-5.8S rDNA-ITS2 region on yeast genomic DNA [25]. Briefly, the ITS region of the isolated genomic DNA was amplified by PCR using a set of universal primers (ITS1 and 4). The primer sequences are as follows: ITS1 (forward, 5′-TCCGTAGGTGAACCTGCGG-3′) and ITS4 (reverse, 5′-TCCTCCGCTTATTGATATGC-3′). PCR was carried out at 95 °C for 5 min (initial denaturation), followed by 33 cycles of denaturation at 95 °C for 1 min, annealing at 55 °C for 1 min, extension at 72 °C for 2 min, and a final extension at 72 °C for 10 min. The PCR amplicons were separated via 1.2% (*w*/*v*) agarose gel electrophoresis, and the targeted DNA bands (0.5–0.8 kb) were isolated and purified using AccuPep PCR/Gel Purification Kit (Bioneer, Daejeon, Republic of Korea). Then, they were sequenced by Biofact Co., Ltd. (Daejeon, Republic of Korea; twice [in forward and reverse directions] for each strain), and the yeast isolates were identified by comparing the nucleotide sequences of the PCR amplicons with those of yeast strains retrieved from GenBank database in the NCBI using BLASTn.

The phylogenetic tree was constructed using DNA sequences of identified yeast species and the MEGAX program based on the neighbor-joining (NJ) method [26].

### 4.4. AOH Standard Solutions, AOH Extraction, Determination of the Levels of AOH by HPLC-UVD, and Validation of the HPLC-UVD Method for AOH Quantification

An AOH stock solution (200 μg/mL) was made by mixing 5 mg of AOH powder with 25 mL of MeOH and stored at −20 °C until use. Five levels of AOH standard solutions (0.1, 0.2, 0.5, 1.0, and 2.0 μg/mL) were prepared freshly by dilution of the stock solution with MeOH.

AOH was extracted from samples by a procedure of Kralova and collaborators with minor modifications [27]. Briefly, each sample was extracted twice with 5 mL of MeOH together with filtration through a GF/A filter paper (Whatman, Maidstone, UK). Then, it was evaporated to dryness under N_2_ at 40 °C and dissolved in 5 mL of 5% NaHCO_3_ solution. After being extracted twice with 5 mL of ethyl acetate, it was dried over 2.5 g of anhydrous sodium sulfate (Na_2_SO_4_) and evaporated to dryness under N_2_ at 40 °C. Subsequently, the residue was dissolved in 0.5 mL of MeOH and filtered through a 0.2 μm syringe filter (Hyundai Micro Co., Ltd., Seoul, Republic of Korea) prior to injection into an HPLC system.

AOH was detected and quantified using an HPLC system (LC-20AT; Shimadzu, Tokyo, Japan) equipped with a UV detector (UVD, SPD-10A, Shimadzu, Tokyo, Japan). The quantification of AOH levels was carried out at 258 nm. Separation was performed on a ZORBAX Eclipse Plus C18 column (5 μm particle size, 4.6 mm × 250 mm; Agilent, Santa Clara, CA, USA) and the column oven temperature was set at 30 °C. The mobile phase consisted of 80% MeOH (MeOH:DW = 80:20, *v*/*v*) containing 300 mg of zinc sulfate (ZnSO_4_·H_2_O)/L of solvent and was pumped into the HPLC system at a flow rate of 0.4 mL/min, giving a total run time of 20 min. The injection volume of the samples was 10 μL.

The linearity of a series of AOH concentrations in the HPLC-UVD method was assessed by a standard curve using five levels of AOH standard solutions (0.1, 0.2, 0.5, 1.0, and 2.0 μg/mL). The calibration curve of AOH was created by plotting the AOH concentrations (x-axis) versus peak areas (y-axis) in HPLC-UVD analysis. The linear regression analysis was then used to evaluate the linearity, which was determined by a coefficient of determination (r^2^).

In order to determine the recovery of AOH, 0.1 M KH_2_PO_4_ buffer solution (pH 8) was spiked with AOH standard solutions (200 μg/mL) to give concentrations of 0.2, 1, and 2 μg/mL AOH. AOH in the fortified samples was extracted according to the procedures described above. After the amounts of AOH extracted from the samples were analyzed by HPLC-UVD, the recoveries were determined using the following equation and shown as mean ± standard deviation.

$$\mathrm{Recovery}=\frac{\mathrm{AOH concentration equivalent to the peak area measured from the spiked sample}\times 100}{\mathrm{AOH concentration used for spiking the sample}}$$

The within-day precision was evaluated by three replicate injections of AOH solutions extracted from the spiked samples within a day and expressed as *RSDr* of AOH levels obtained in triplicate.

The LOQ and LOD were used to assess the sensitivity of the analytical method using HPLC-UVD. Those values were calculated by the following equation using the slope of the standard curve and the standard deviation of the response.

$$\mathrm{LOQ}=\frac{10\times \mathrm{standard deviation}}{\mathrm{slope}}$$

$$\mathrm{LOD}=\frac{3.3\times \mathrm{standard deviation}}{\mathrm{slope}}$$

### 4.5. Screening for AOH-Detoxifying Yeasts

An overnight seed culture (100 μL, OD_600nm_ = 0.1) of each yeast strain was inoculated into 5 mL of 0.1 M KH_2_PO_4_ buffer solution (pH 8) spiked with 1 μg/mL of AOH and incubated at 30 °C under agitation at 150 rpm for 48 h. Sterile DW (100 μL) instead of yeast seed culture was used as a control. After the cultured samples were centrifuged at 4000 rpm for 10 min, AOH was extracted from the supernatant and analyzed by the procedure using HPLC as described above. AOH reduction rates were calculated and expressed as mean ± standard deviation.

### 4.6. AOH Reduction Rates by R. taiwanensis P1 Under Different Culture Conditions

The influence of incubation temperature and incubation time on AOH reduction rates by yeasts was tested using phosphate buffer solution (pH 8). To evaluate the impact of incubation time, 100 μL of yeast cell suspension (OD_600nm_ = 0.1) was inoculated into 5 mL of 0.1 M KH_2_PO_4_ buffer solution (pH 8) spiked with 1 μg/mL of AOH after the yeast strain was cultured in YPD at 30 °C under agitation at 150 rpm overnight. Then, it was incubated at 30 °C under agitation at 150 rpm for different culture times (0, 6, 12, 24, 36, 48, and 72 h). Five mL of the buffer solution spiked with AOH (1 μg/mL) was used as a control after sterile DW (100 μL) was added to it.

To assess the impact of incubation temperature, 5 mL of 0.1 M KH_2_PO_4_ buffer solution (pH 8) spiked with 1 μg/mL of AOH was incubated at 25, 30, 35, and 40 °C under agitation at 150 rpm for 48 h after inoculation of yeast seed culture (100 μL, OD_600nm_ = 0.1). Five mL of the buffer solution spiked with AOH (1 μg/mL) was used as a control after sterile DW (100 μL) was added to it.

After the cultured samples were centrifuged at 4000 rpm for 10 min, AOH was extracted from the supernatant, and the level of AOH was determined by HPLC-UVD as described above.

### 4.7. Mechanism of AOH Detoxification by R. taiwanensis P1

For AOH detoxification test using inactivated cells (dead cells), yeast cell suspension (OD_600nm_ = 0.1) was autoclaved at 121 °C for 15 min. Then, 5 mL of 0.1 M KH_2_PO_4_ buffer solution (pH 8) spiked with 1 μg/mL of AOH was incubated at 30 °C under agitation at 150 rpm for 48 h after inoculation of autoclaved yeast cell suspension (100 μL) into the buffer solution. Five mL of the buffer solution spiked with AOH (1 μg/mL) was used as a control after sterile DW (100 μL) was added to it. Then, after the cultured samples were centrifuged at 4000 rpm for 10 min, AOH was extracted from the supernatant, which was followed by HPLC analysis as described above.

For AOH detoxification test using yeast cell-free filtrate, the filtrate was prepared as follows. After yeast cell suspension (OD_600nm_ = 0.1) was centrifuged at 4 °C and 4000 rpm for 10 min, the culture supernatant (100 μL) was added to 5 mL of 0.1 M KH_2_PO_4_ buffer solution (pH 8) spiked with 1 μg/mL of AOH. It was then incubated at 30 °C under agitation at 150 rpm for 48 h. Five mL of the buffer solution (pH 8) spiked with 1 μg/mL of AOH was used as a control after sterile DW (100 μL) was added to it. Then, AOH was extracted from the samples, and the amount of AOH was determined by HPLC analysis as described above.

For the AOH detoxification test using intracellular enzymes from yeast cells, the intracellular enzyme fraction was isolated as follows. After inoculation of yeast cell suspension (100 μL, OD_600nm_ = 0.1) into 5 mL of 0.1 M KH_2_PO_4_ buffer solution (pH 8), it was incubated at 30 °C under agitation at 150 rpm for 48 h. Then, it was centrifuged at 4 °C and 4000 rpm for 10 min, and yeast cell pellets were resuspended in the buffer solution. After the yeast cells were ground under liquid N_2_, the disrupted yeast cells were resuspended in the phosphate buffer (pH 8). After being centrifuged at 4000 rpm for 10 min, the supernatant was used as the intracellular enzyme fraction, whereas the cell debris pellet was isolated as the cell wall fraction by resuspension in the phosphate buffer (pH 8). Next, 100 μL of the intracellular enzyme fraction was added to 5 mL of 0.1 M KH_2_PO_4_ buffer solution (pH 8) spiked with 1 μg/mL of AOH, and it was incubated at 30 °C under agitation at 150 rpm for 48 h. Five mL of 0.1 M KH_2_PO_4_ buffer solution (pH 8) spiked with 1 μg/mL of AOH was used as a control after phosphate buffer (100 μL) was added to it. AOH was then extracted from the samples, followed by HPLC analysis as described above.

In order to test whether AOH was bound to the yeast cell wall, the yeast cell wall fraction, which was isolated as described above, was washed twice with sterile DW and resuspended in 5 mL of 0.1 M KH_2_PO_4_ buffer solution (pH 8) spiked with 1 μg/mL of AOH. After it was incubated at 30 °C under agitation at 150 rpm for 48 h and centrifuged at 4 °C and 4000 rpm for 10 min, AOH was extracted from the supernatant and the yeast cell wall pellet, which was followed by HPLC analysis as described above.

For the AOH detoxification test using yeast spheroplasts, after yeast culture in 0.1 M KH_2_PO_4_ buffer solution (pH 8) at 30 °C and 150 rpm for 48 h as described above, the spheroplasts were generated by treatment of yeast cell suspension supplemented with 1 M sorbitol with lyticase (0.5 mg/mL) at 37 °C for 1 h. The spheroplast pellet was resuspended in 0.5 mL of phosphate buffer (pH 8) supplemented with 1 M sorbitol after centrifugation at 4 °C and 4000 rpm for 10 min, and it was added to 4.5 mL of 0.1 M KH_2_PO_4_ buffer solution (pH 8) spiked with 1 μg/mL of AOH and 1 M sorbitol. Then, it was incubated at 30 °C under agitation at 150 rpm for 48 h. Phosphate buffer (5 mL, pH 8) fortified with 1 μg/mL of AOH and 1 M sorbitol was used as a control. AOH was then extracted from the supernatant after centrifugation at 4 °C and 4000 rpm for 10 min, followed by HPLC analysis as described above.

### 4.8. Statistical Analyses

Statistical analyses were performed by a one-way analysis of variance (ANOVA) using SPSS 29.0 (SPSS Inc., Chicago, IL, USA), which was followed by Tukey’s multiple range comparison as a post-hoc test. The results were represented as the mean ± standard deviation and were considered to be statistically different at a *p* value < 0.05.

## 5. Patents

The authors declare that a patent based on this study has been granted (Korea Patent No. 10-2961284). *Rhodotorula taiwanensis* P1 was deposited in the Korean Agricultural Culture Collection (KACC) under the strain number KACC 93448P.

## Figures and Tables

**Table 1:** Recoveries and precision of AOH in KH_2_PO_4_ buffer solution (pH 8).

AOH Level (μg/mL)	Recovery (%)	*RSDr* ^1^ (%)
0.2	92.01 ± 1.83	1.99
0.5	98.76 ± 7.94	8.04
1.0	100.33 ± 6.28	6.26

^1^ RSDr represents relative standard deviation generated from results obtained under within-day precision conditions. Recovery and *RSDr* were analyzed in triplicate. Data are represented as the mean ± standard deviation.

**Table 2:** Identification of 10 yeast isolates from pears, grapes, and strawberries using BLASTn-based analysis.

Sample ID	Isolate or Strain No. from NCBI	Scientific Name	Order (Phylum)	Sequence Similarity (%)
P1	MGLH-11 KACC 49635	*Rhodotorula taiwanensis*	Sporidiobolales (Basidiomycota)	99.61
P2	CBS 6020	*Rhodotorula babjevae*	Sporidiobolales (Basidiomycota)	99.98
P3	CBS 4412	*Kurtzmaniellla quercitrusa*	Serinales (Saccharomycota)	99.20
P4	12	*Cystobasidium slooffiae*	Cystobasidiales (Basidiomycota)	99.47
P5	NS-O-109	*Aureobasidium pullulans*	Dothideales (Ascomycota)	99.23
G1	CBS 2030	*Meyerozyma guilliermondii*	Saccharomycetales (Ascomycota)	99.52
G2	7z-11	*Zygotorulapspora Florentina*	Saccharomycetales (Ascomycota)	99.94
G3	MCZ 43	*Rhodotorula mucilaginosa*	Sporidiobolales (Basidiomycota)	99.76
S1	MCA 3686	*Sporidiobolus pararoseus*	Sporidiobolales (Basidiomycota)	99.49
S2	HAI-Y-308	*Cryptococcus albidus*	Tremellales (Basidiomycota)	99.34

BLASTn was run using ITS1-5.8S rDNA-ITS2 sequences.

**Table 3:** AOH reduction rates of yeast isolates after culture in 0.1 M KH_2_PO_4_ buffer solution (pH 8) spiked with 1 μg/mL of AOH at 30 °C under agitation at 150 rpm for 48 h.

Yeast Isolate	AOH Reduction Rate (%)
*Rhodotorula taiwanensis* P1	76.87 ± 1.59 ^a^
*Rhodotorula babjevae* P2	53.40 ± 1.32 ^b^
*Kurtzmaniellla quercitrusa* P3	37.77 ± 0.40 ^c^
*Cystobasidium slooffiae* P4	22.57 ± 2.21 ^d^
*Aureobasidium pullulans* P5	22.63 ± 1.58 ^d^
*Meyerozyma guilliermondii* G1	31.83 ± 0.42 ^e^
*Zygotorulapspora florentina* G2	21.73 ± 3.10 ^d^
*Rhodotorula mucilaginosa* G3	22.53 ± 0.67 ^d^
*Sporidiobolus pararoseus* S1	22.43 ± 2.57 ^d^
*Cryptococcus albidus* S2	22.67 ± 0.93 ^d^
Control (sterile DW without yeast)	23.37 ± 1.83 ^d^

Different letters within the same column indicate significant differences (*p* < 0.05). All data were obtained in triplicate and are represented as the mean ± standard deviation.

## Data Availability

The original contributions presented in this study are included in the article. Further inquiries can be directed to the corresponding author.

## Abbreviations

The following abbreviations are used in this manuscript:

AOH	Alternariol
AME	Alternariol monomethyl ether
TeA	Tenuazonic acid
